# Understanding facilitators of research participation among adults with self-reported chronic pain – a survey examining hypothetical research participation

**DOI:** 10.1186/s12874-023-02128-8

**Published:** 2024-01-22

**Authors:** Charles Start, Meagan McBride, Guohao Zhu, Sana Shaikh, Jennifer Pierce

**Affiliations:** Department of Anesthesiology, University of Michigan, Back & Pain Center, 325 E. Eisenhower Parkway, Burlington Building 1, Suite 310, Ann Arbor, MI 48108 USA

**Keywords:** Chronic pain, Research participation, Study recruitment, Motives for research participation, Participant preference

## Abstract

**Background:**

An inability to successfully recruit participants into clinical research has consequences that negatively affect the conduct and reliability of research studies. Understanding facilitators of research participation, namely motives for participation and preferred research outcomes, may improve recruitment and retention of clinical trials related to chronic pain. The present study explored research participation facilitators among individuals with chronic pain and their association with demographic characteristics, pain-related characteristics, and factors related to future research engagement.

**Methods:**

Individuals from Michigan who were 18 years or older and self-reported having chronic pain completed an online survey assessing motives for research participation and desired research outcomes. Analyses were conducted in three stages. First, we evaluated underlying factors of motives for participation and research outcome preferences using principal components analysis. Second, we classified individuals according to their patterns of facilitators using latent profile analysis. Finally, we evaluated differences between facilitator profiles in demographic characteristics, pain-related characteristics, and factors related to future research engagement using χ^2^ analyses and Kruskal-Wallis rank sum tests.

**Results:**

Three components of motives for research participation were identified: social engagement/enjoyment; pain improvement/advancing science; and compensation. Three components of research outcome preferences were identified: co-occurring symptom reduction; behavior reduction modification; and pain and function improvement. Four potential patient-centered profiles utilizing these dimensions of facilitators were identified that had unique demographic characteristics, research participation willingness, and treatment interest.

**Conclusions:**

Our data provide a framework of motives and research outcome preferences that may inform recruitment and retention in chronic pain research. It also gives an indication of who may respond best to active or passive recruitment strategies that appeal to a given motive or preferred outcome. This information may be useful for improving recruitment and to monitor any potential biases in participant samples.

**Supplementary Information:**

The online version contains supplementary material available at 10.1186/s12874-023-02128-8.

## Introduction

Participation in clinical trials is integral to the development of new knowledge that can be implemented in clinical practice. An inability to successfully recruit participants into clinical research studies poses numerous problems, including wasted resources, biased findings, and lack of statistical power to find meaningful effects [1]. Recruitment challenges may be amplified for studies evaluating individuals with chronic pain given the unique and far-reaching impact that pain has on individuals’ social, psychological, and physical functioning [2]. Although most studies focus on reducing barriers to improve recruitment efforts, appealing to individuals’ values, motives, and desires may be another valuable tool [1]. Indeed, great importance has been placed on conducting patient-centered, meaningful research through the engagement of patients and other stakeholders in the research process, which is expected to enhance recruitment and retention [3–5]. The present study focuses on motives for research participation and research outcome preferences as facilitators to engagement.

The existing literature on motives for participating in research tends to focus on specific groups of people or disease populations. A review of this literature found that participants’ motives for engaging in research are influenced by the disease being studied [6]. A qualitative study by Wasan et al. [7] examined research motives specifically for patients with chronic pain and found that individuals often cited both altruistic and personal motives. Altruistic motives included contributing to research and helping others with their pain, while personal benefits included money, learning about and trying different treatments, and relief from pain. Importantly, however, motives for research participation are complex, interrelated, and not solely determined by the disease of interest [6, 8]. Instead, a person-centered approach may reveal different underlying patterns of motives for research participation that enhance our ability to understand and apply this information to improve research goals and processes.

Considering outcome preferences alongside motives for research participation may provide a more complete picture of facilitators to research engagement in the chronic pain population. In addition to relief from pain, other outcomes exist that chronic pain patients want researchers to focus on. Such outcomes include improving enjoyment of life, physical function, emotional well-being, fatigue, weakness, and sleep related problems [9, 10]. Designing research that focuses on outcomes that are relevant and meaningful to patients will presumably enhance participation and engagement resulting in downstream benefits on retention and adherence [5].

To our knowledge, no research exists that explores patterns of motives for research participation and research outcome preferences among individuals with chronic pain and their association with demographic and pain characteristics. Thus, the exploratory and hypothesis-generating aims of the present study were three-fold. First, we sought to explore underlying dimensions of motives for hypothetical research participation and research outcome preferences among individuals with self-reported chronic pain. Second, we took a person-oriented approach to determine potential patterns of motives for hypothetical research participation and research outcome preferences. Finally, we explored whether these patterns of motives for research participation and research outcome preferences are associated with participant characteristics, willingness to engage in future research, and treatment interest.

## Methods

### Participants and procedure

Participants were recruited through advertisements on social media platforms, such as Facebook and Reddit, to complete a single, online, anonymous survey. The study design was therefore cross-sectional. The survey was hosted via Qualtrics and interested individuals were able to click a link to complete an online inclusion/exclusion screener and informed consent. If all criteria were met and informed consent was indicated, individuals proceeded to the survey. Targeted social media advertisements were posted from January 2021 to April 2022, spanning chronic pain social media sites, veterans’ sites, and community sites across Michigan. Detailed information regarding the advertising technique is available as supplemental material (see Supplemental Fig. 1 and Supplemental Tables 1–3). Participants were required to be 18 years of age or older, live in the state of Michigan, and self-report chronic pain (i.e., any persistent or recurrent body pain for the last 3 months or longer). The study sample was restricted to Michigan residents because the findings were intended to improve research recruitment processes at the Back & Pain Center at the University of Michigan, which typically serves patients from Michigan. All methods were carried out in accordance with relevant guidelines and regulations. The study was approved by the Michigan Medicine Institutional Review Board (HUM00209650). Upon completion of the survey, participants had the option to complete an additional, unlinked survey for 10 USD remuneration.

### Measures

#### Motives for hypothetical research participation

Participants completed 14 items assessing factors that would make them want to participate in research. Items were based on identified psychosocial facilitators and barriers to research participation, mapped to the Theoretical Domains Framework (TDF) as well as prior qualitative interview-based research [6, 11]. They were asked: “We would like to know what would make you want to participate in research. Please think about the reasons you have participated in research in the past and what would make you want to do so in the future. There are no right or wrong answers – this is just what motivates you. Please indicate how much you agree with the following statements. I would participate in research…” Sample items include: “To improve the wellbeing of society” and “To receive compensation.” Items are listed in detail in Table 1. Response options included 1 (Not at all) to 5 (Completely).

#### Research outcome preferences

Participants completed 13 items assessing the subjective importance of various pain research treatment outcomes. They were asked: “Many researchers are interested in studying a certain outcome. An outcome is some kind of symptom or behavior that the researcher hopes to see change as a result of the treatment. What types of outcomes would be most important for you? In other words, what treatment results would you like pain research to focus on? How important do you consider the following outcomes?” Sample items include: “Lower severity or intensity of pain” and “Better sleep”. Items are listed in detail in Table 2. Response options included 1 (Not at all) to 5 (Extremely).

#### Demographics

Age, gender, race, income, education, and employment status were assessed. Gender was recoded to include 0 (Male); 1 (Female); and 2 (Transgender or Non-Binary). Race was recoded to include 0 (Non-White) and 1 (White). Income was recoded to include 0 (Greater than or equal to 50,000 USD per year) and 1 (Less than 50,000 USD per year). Education was recoded to include 0 (Less than Associate’s Degree) and 1 (Associate’s Degree or higher education). Employment status was recoded to include 0 (Not currently working part- or full-time) and 1 (Currently working part- or full-time).

#### Pain duration

Participants were asked to indicate how long they had chronic pain, with response options ranging from 1 (3 months or less) to 7 (6 or more years). Scores were dichotomized to indicate 0 (Less than 1 year) and 1 (One year or more).

#### Pain severity

The 4-item Brief Pain Inventory was used to assess pain severity [12]. Participants were asked to rate their pain at its worst in the last week, least in the last week, on average, and right now, with options ranging from 0 (No pain) to 10 (Pain as bad as you can imagine). An average score was calculated.

#### Physical function

The 4-item Patient-Reported Outcomes Measurement Information System (PROMIS) Physical Function – Short Form 4a was used to assess physical function [13]. Sample items include: “Are you able to do chores such as vacuuming or yard work?” and “Are you able to go up and down stairs at a normal pace?” Response options included 1 (Unable to do) to 5 (Without any difficulty). A sum score was obtained.

#### Depressive and anxiety symptoms

Brief, 2-item measures were used to assess depressive symptoms (Patient Health Questionnaire-2; [14]) and anxiety symptoms (Generalized Anxiety Disorder-2; [15]). Participants were asked to consider how often they were bothered by symptoms over the last 2 weeks. Items to assess depressive symptoms included: “Little interest or pleasure in doing things” and “Feeling down, depressed, or hopeless.” Items to assess anxiety symptoms included: “Feeling nervous, anxious or on edge” and “Not being able to stop or control worrying.” Response options included 0 (Not at all) to 3 (Nearly every day). An average score was obtained for each 2-item measure.

#### Perceived health

A single item was used to assess perceived health [16]. Participants were asked: “In general, would you say your health is…” Response options included 1 (Excellent) to 5 (Poor).

#### Willingness to participate in future research

Ten items were used to determine participants’ willingness to participate in future research. Participants were asked to consider how willing they would be to participate in a study that included various requirements, including, for example, “A one-time survey online” and “A medication”. Response options included 1 (Not at all) to 5 (Definitely).

#### Treatment preferences

Participants were presented with 16 potential pain interventions and asked to indicate which treatments they would be interested in trying and which treatments they would refuse to try. Examples of treatments included “Acupuncture/Acupressure”, “Mental health therapy or counseling”, and “Opioid medication”.

### Data analyses

All analyses were conducted using R 4.2.1 [17] and proceeded in three stages outlined below. Because the survey was anonymous and online, data cleaning was conducted to ensure that the responses were valid. Initially, 1,170 individuals clicked on the survey link. Of these, 691 individuals were eligible following the inclusion and exclusion criteria. We dropped cases in which less than 50% of the survey was completed (n = 157) and the survey completion time was less than 5 min (n = 4). We then scanned the data for questionable response patterns, including strings of responses across scales that included reverse-coded items and nonsense open-ended responses. Using a conservative approach, an additional 47 responses were removed from the dataset. The final dataset included 483 observations. However, the present study limited the sample to individuals who completed items related to motives for hypothetical research participation and research outcome preferences (n = 412).

#### Principal components analysis

Means and standard deviations were assessed for all motives and preference items. Bivariate correlations were also evaluated among each set of items. Principal axis factoring with orthogonal rotation (i.e., varimax) was performed. Oblique rotation yielded similar results; thus, for interpretability, we report only the varimax rotation. The number of factors was determined according to the scree plot, variance explained, and interpretability. Factor loadings were assessed in the corresponding factor matrices. Items that cross-loaded on more than one dimension with a factor loading > 0.3 were considered for deletion; item removal was performed iteratively. After the factor structure was determined, subscales were created by averaging the items and reliability was assessed. Bivariate correlations among the subscales were also evaluated.

#### Latent profile analysis

Patterns of motives and preferences were then evaluated using latent profile analysis. The mclust package (version 5.4.10) from R was used to conduct the latent profile analysis. The mclust package includes functions for model-based clustering based on parameterized finite Gaussian mixture models. Standardized indicators were fitted by models with different covariance structures and different numbers of profiles. A plot of Bayesian Information Criteria for all the models with profiles ranging from 1 to 9 was generated to select the optimal model. After determining the best fitting model, participants were then assigned to a single group according to their highest probability score. Means and standard deviations on motive and preference subscales were then evaluated for each group.

#### Group differences

Group differences in demographic characteristics, pain-related characteristics, willingness to participate in future research, and interest in various treatments were then evaluated. For categorical variables, Pearson’s χ^2^ tests were assessed to determine omnibus effects. Fisher’s Exact Test was used where expected cell counts were less than five. For continuous variables, Kruskal-Wallis rank sum test was conducted. To control for Type I error due to multiple tests, a Bonferroni correction was applied such that significance was determined according to $\alpha$< 0.05/60 (*p* < .00083) for all omnibus significance tests. For continuous variables, Games-Howell post-hoc tests were assessed to evaluate the pairwise differences in averages where omnibus *p* values were < 0.00083. Data, analysis code, and output are available on Open Science Framework for transparency (https://osf.io/8m5gs/?view_only=f0637c6e9b4748209f5d6a67f1652355). The Checklist for Reporting Of Survey Studies (CROSS) has been used for reporting this study and is provided as supplemental material (see Supplemental Table 4).

## Results

### Sample characteristics

The final sample included 412 participants (M_age_ = 40.85, SD = 15.85). The majority of the sample identified as female (52%), with 43% identifying as male and 5% identifying as transgender or non-binary. Most of the sample identified as White (61%).

### Principal components analyses

#### Factor structure of motives for hypothetical research participation

Raw correlations among the original items assessing motives for hypothetical research participation are presented in Supplemental Table 5. The factor structure of the original 14 items yielded three factors with an eigenvalue over 1, which was confirmed by the scree plot and evaluation of interpretability of the components. The cumulative percentage of variance explained by the three factors was 58%. As seen in Table 1, four items indicated cross-loading > 0.3 on more than one factor. These items were removed in an iterative process, with each step revealing similar cross-loading of the remaining problematic items. The final model retained a three-factor structure with 65% cumulative variance explained. The items included in the final model are presented in Table 1. The first 4-item factor focused on social engagement and enjoyment. The second 4-item factor focused on pain improvement and advancing science. The third and final factor included two items focused on compensation.

**Table 1:** Factor structure of motives for hypothetical research participation (n = 412)

		Original Factor Loadings			Final Model Factor Loadings		
I would participate in research…	M (SD)	1	2	3	1	2	3
To improve the wellbeing of society.	3.64 (1.00)	− 0.05	0.71	− 0.04	− 0.04	**0.73**	− 0.06
To improve my quality of life and health.	3.95 (0.94)	− 0.09	0.82	0.13	− 0.09	**0.83**	0.08
To learn about my pain.	3.90 (1.02)	0.14	0.74	0.15	0.16	**0.78**	0.10
To have access to new treatments.	4.00 (1.00)	0.07	0.71	0.35	0.07	**0.73**	0.29
To have faster and more available access to doctors.	3.67 (1.05)	0.10	0.48	0.58	--	--	--
To receive medical care without insurance/payment.	3.23 (1.24)	0.10	0.08	0.83	0.12	0.16	**0.84**
To receive compensation.	3.29 (1.23)	0.34	0.05	0.64	0.30	0.09	**0.77**
If it was recommended by my doctor.	3.45 (1.18)	0.36	0.36	0.41	--	--	--
If it was recommended by family or friends.	3.20 (1.24)	0.60	0.20	0.36	--	--	--
If it’s easy for me to do.	3.70 (1.04)	0.44	0.55	0.02	--	--	--
If a lot of people I know do it.	2.68 (1.30)	0.75	− 0.14	0.26	**0.74**	− 0.12	0.25
If it is fun.	2.85 (1.19)	0.80	0.10	0.10	**0.80**	0.11	0.17
To share my story.	3.20 (1.19)	0.69	0.22	0.01	**0.75**	0.27	− 0.01
To fill my time.	2.50 (1.27)	0.74	− 0.19	0.19	**0.77**	− 0.17	0.17
*Percent of common variance explained*		22%	22%	14%	25%	25%	15%
*Cumulative variance explained*				58%			65%

*Note*: Boldfaced values in the final model indicate proposed components based on highest factor loadings

#### Factor structure of research outcome preferences

Raw correlations among the original items assessing research outcome preferences are presented in Supplemental Table 6. The factor structure of the original 13 items revealed two factors with an eigenvalue over 1; however, the scree plot and assessment of interpretability favored a three-factor solution. The cumulative variance explained by the three factors was 63%. As seen in Table 2, one item that had similar loadings on two factors was removed. The final model included three factors with 63% cumulative variance explained. The items included in the final model are presented in Table 2. Although several items showed cross-loading > 0.3, one loading was notably larger. Therefore, the items were retained, and they were assigned to the highest loading factor. The first 6-item factor focused on co-occurring symptom reduction. The second 2-item factor focused on behavior reduction modification. The third and final factor included four items and focused on pain and function improvement.

**Table 2:** Factor structure of research outcome preferences

		Original Factor Loadings			Final Model Factor Loadings		
How important do you consider the following outcomes:	M (SD)	1	2	3	1	2	3
Lower severity or intensity of pain	3.90 (0.96)	0.44	0.70	− 0.16	0.48	− 0.19	**0.67**
Better physical functioning	3.88 (1.01)	0.47	0.66	− 0.02	0.52	− 0.03	**0.61**
Learning new ways to manage pain	3.94 (0.98)	0.25	0.76	0.20	0.28	0.17	**0.76**
Increased frequency of healthy behaviors like exercise	3.67 (1.00)	0.09	0.70	0.41	0.11	0.37	**0.73**
Decreased frequency of behavior like drinking or smoking	2.90 (1.33)	0.04	0.06	0.80	0.08	**0.84**	0.02
Better mood (like lower depression or anxiety symptoms)	3.62 (1.08)	0.65	0.15	0.33	**0.68**	0.35	0.09
Better sleep	3.84 (1.05)	0.65	0.36	0.12	**0.70**	0.14	0.29
Less fatigue	3.85 (1.06)	0.72	0.34	− 0.03	**0.76**	0.00	0.26
Better mental clarity and concentration	3.77 (1.08)	0.72	0.14	0.28	**0.71**	0.27	0.14
Increased ability to enjoy life	3.91 (1.04)	0.72	0.32	0.14	**0.72**	0.12	0.31
Better relationships, such as with friends or a romantic partner	3.59 (1.16)	0.61	− 0.01	0.57	--	--	--
Better overall well-being	3.91 (1.01)	0.71	0.33	0.13	**0.71**	0.12	0.31
Reducing or stopping a medication	3.54 (1.21)	0.25	0.15	0.64	0.24	**0.65**	0.18
*Percent of common variance explained*		29%	19%	14%	31%	13%	20%
*Cumulative variance explained*				63%			63%

*Note:* Boldfaced values in the final model indicate proposed components based on highest factor loadings

#### Associations between factors

As shown in Table 3, bivariate correlations suggest that social and enjoyment motives for research were moderately associated with compensation motives, and research preferences related to behavior reduction modification. Motives related to pain improvement and advancing science were moderately associated with research preferences related to co-occurring symptom reduction and pain and function improvement. Research preferences related to co-occurring symptom reduction were moderately associated with preferences related to behavior reduction modification and strongly associated with preferences related to pain and function improvement. This strong association was also suggested by the strong cross-loading of pain-related items on the factor representing co-occurring symptoms. Thus, those who indicate research preferences related to co-occurring symptom reduction also indicate strong preferences related to pain and function improvement.

**Table 3:** Bivariate correlations among subscales

		Reliability	1	2	3	4	5
1	Motives Factor 1: **Social Engagement and Enjoyment**	0.79	--				
2	Motives Factor 2: **Pain Improvement and Advancing Science**	0.78	0.08	--			
3	Motives Factor 3: **Compensation**	0.46^a^	0.42	0.27	--		
4	Preference Factor 1: **Co-Occurring Symptom Reduction**	0.87	0.00	0.52	0.20	--	
5	Preference Factor 2: **Behavior Reduction Modification**	0.31^a^	0.30	0.19	0.27	0.39	--
6	Preference Factor 3: **Pain and Function Improvement**	0.79	− 0.14	0.53	0.11	0.69	0.29

*Note:* Cronbach’s α is reported for reliability. ^a^For subscales containing only two items, Pearson product-moment correlations are reported

### Participant-centered patterns of motives and interests

Latent profile analysis favored four clusters of participants. As shown in Table 4, Group 1 (n = 106) was characterized by low pain-related facilitators. Specifically, this group was not particularly high on any factor; however, they did show comparatively low scores on motives for research participation related to pain improvement and advancing science as well as comparatively low scores on research outcome preferences related to co-occurring symptom reduction and pain and function improvement. Group 2 (n = 112) was comparatively high on all motives for research participation and all research outcome preferences, suggesting a general interest in research. Group 3 (n = 128) was not particularly high on any factor but did show comparatively low scores on research outcome preferences related to behavior reduction modification. Finally, Group 4 (n = 66) was particularly high on motives related to pain improvement and advancing science and research outcome preferences related to co-occurring symptom reduction and pain and function improvement. However, they were very low on motives related to social engagement and enjoyment.

**Table 4:** Differences in Motives and Preferences for LPA Groups

			Group 1: Low Pain-Related Facilitators	Group 2: High Motivation and Interest	Group 3: Low Behavior Reduction Outcome Preference	Group 4: High Pain-Related Facilitators, Low Social Motives	
		Overall	n = 106	n = 112	n = 128	n = 66	*p*
Motives for Research Participation							
	Motives Factor 1: **Social Engagement and Enjoyment**	2.81 (0.97)	3.08 (0.49)	3.70 (0.50)	2.32 (0.95)	1.80 (0.55)	< 0.001
	Motives Factor 2: **Pain Improvement and Advancing Science**	3.87 (0.77)	3.13 (0.46)	4.23 (0.47)	3.83 (0.81)	4.54 (0.43)	< 0.001
	Motives Factor 3: **Compensation**	1.63 (0.53)	1.62 (0.34)	1.83 (0.44)	1.38 (0.62)	1.80 (0.50)	< 0.001
Research Outcome Preferences							
	Preference Factor 1: **Co-Occurring Symptom Reduction**	3.81 (0.81)	3.10 (0.37)	4.24 (0.55)	3.67 (0.89)	4.54 (0.43)	< 0.001
	Preference Factor 2: **Behavior Reduction Modification**	3.22 (1.03)	3.10 (0.58)	4.02 (0.57)	2.25 (0.89)	3.91 (0.78)	< 0.001
	Preference Factor 3: **Pain and Function Improvement**	3.85 (0.78)	3.02 (0.42)	4.17 (0.53)	3.94 (0.77)	4.46 (0.47)	< 0.001

*Note:* All tests are significant at *p* < .00083

### Associations with demographic and pain-related characteristics

As shown in Table 5, Group 1 (individuals with low pain-related facilitators) and Group 2 (individuals with high motivation and interest) were similar on various characteristics, whereas Group 3 (individuals with low behavior reduction outcome preference) and Group 4 (individuals with high pain-related facilitators but low social motives) were similar. Groups 1 and 2 were generally younger, more often male, less often White, more often employed, and had pain of shorter duration. Groups 3 and 4 were generally older, more often female, primarily White race, more often unemployed and had pain of longer duration. Group 1 additionally indicated comparatively high depressive symptoms.

**Table 5:** Differences in Patient Characteristics Among Motives and Preference Groups

		Overall	Group 1: Low Pain-Related Facilitators n = 106	Group 2: High Motivation and Interest n = 112	Group 3: Low Behavior Reduction Outcome Preference n = 128	Group 4: High Pain-Related Facilitators, Low Social Motives n = 66	*p*
Age		40.85 (15.85)	**31.80 (7.73)** ^**a**^	**33.80 (10.94)** ^**a**^	**50.63 (17.77)** ^**b**^	**48.35 (14.38)** ^**b**^	**< 0.001**
Gender							
	Male	43% (176)	**71% (75)**	**60% (67)**	**20% (26)**	**12% (8)**	**< 0.001**
	Female	52% (215)	**19% (20)**	**38% (42)**	**77% (98)**	**83% (55)**	
	Transgender/Non-binary	5% (21)	**10% (11)**	**2% (3)**	**3% (4)**	**5% (3)**	
Race							
	White	61% (252)	**48% (51)**	**45% (50)**	**80% (102)**	**74% (49)**	**< 0.001**
	Non-White	39% (160)	**52% (55)**	**55% (62)**	**20% (26)**	**26% (17)**	
Income							
	< $50,000 per year	37% (151)	42% (44)	29% (32)	35% (45)	45% (30)	0.088
	≥ $50,000 per year	63% (261)	58% (62)	71% (80)	65% (83)	55% (36)	
Education							
	Less than college education	35% (146)	46% (49)	34% (38)	28% (36)	35% (23)	0.037
	Some college or higher education	65% (266)	54% (57)	66% (74)	72% (92)	65% (43)	
Employment							
	Employed	71% (273)	**92% (96)**	**85% (91)**	**48% (55)**	**51% (31)**	**< 0.001**
	Unemployed, retired, or student	29% (113)	**8% (8)**	**15% (16)**	**52% (59)**	**49% (30)**	
Pain duration							
	Pain lasting less than one year	47% (195)	**88% (93)**	**62% (69)**	**21% (27)**	**9% (6)**	**< 0.001**
	Pain lasting over one year	53% (217)	**12% (13)**	**38% (43)**	**79% (101)**	**91% (60)**	
Pain severity		4.62 (1.69)	4.66 (1.72)	4.70 (1.86)	4.45 (1.62)	4.74 (1.49)	0.400
Physical function		13.79 (3.73)	13.08 (2.86)	14.70 (3.26)	13.34 (4.50)	14.29 (3.76)	0.001
Depressive symptoms		1.10 (0.77)	**1.38 (0.50)** ^**a**^	**1.11 (0.70)** ^**b**^	**0.89 (0.91)** ^**b**^	**1.07 (0.82)** ^**b**^	**< 0.001**
Anxiety symptoms		1.21 (0.87)	1.43 (0.65)	1.21 (0.86)	1.07 (0.99)	1.14 (0.91)	0.001
Perceived health		3.42 (0.96)	3.44 (0.97)	3.37 (1.01)	3.34 (0.95)	3.61 (0.86)	0.300

*Note:* Boldfaced values displayed omnibus significance at *p* < .00083. Different superscripts denote significant differences between groups on continuous variables according to post-hoc Games-Howell tests

### Associations with future research engagement

As shown in Table 6, individuals in Group 1 (individuals with low pain-related facilitators) were less willing to participate in various types of studies compared to other groups. Individuals in Group 4 (individuals with high pain-related facilitators but low social motives) were generally most willing to engage in various types of studies compared to other groups.

**Table 6:** Differences in Willingness to Participate in Future Research Among Motives and Preference Groups

	Overall	Group 1: Low Pain-Related Facilitators n = 106	Group 2: High Motivation and Interest n = 112	Group 3: Low Behavior Reduction Outcome Preference n = 128	Group 4: High Pain-Related Facilitators, Low Social Motives n = 66	*p*
One-time survey online	3.98 (1.07)	**3.03 (0.90)** ^**a**^	**4.21 (0.92)** ^**b**^	**4.27 (0.96)** ^**bc**^	**4.55 (0.75)** ^**c**^	**< 0.001**
One-time survey on paper	3.44 (1.27)	**2.76 (1.05)** ^**a**^	**3.19 (1.28)** ^**b**^	**3.85 (1.16)** ^**c**^	**4.15 (1.14)** ^**c**^	**< 0.001**
Multiple surveys over time	3.49 (1.20)	**3.05 (0.95)** ^**a**^	**3.28 (1.24)** ^**a**^	**3.69 (1.21)** ^**b**^	**4.15 (1.09)** ^**c**^	**< 0.001**
One-time in-person visit	3.06 (1.24)	**2.81 (1.02)** ^**a**^	**2.81 (1.26)** ^**a**^	**3.13 (1.31)** ^**a**^	**3.74 (1.14)** ^**b**^	**< 0.001**
Multiple in-person visits	2.67 (1.23)	2.75 (0.96)	2.43 (1.34)	2.57 (1.29)	3.12 (1.22)	0.001
Some mild discomfort (such as putting your hand in cold water)	3.17 (1.14)	**2.89 (0.88)** ^**a**^	**2.92 (1.16)** ^**a**^	**3.34 (1.19)** ^**b**^	**3.71 (1.17)** ^**b**^	**< 0.001**
Taking pictures of your brain (such as with MRI)	3.01 (1.24)	**2.76 (0.92)** ^**a**^	**2.78 (1.26)** ^**ab**^	**3.16 (1.28)** ^**bc**^	**3.55 (1.37)** ^**c**^	**< 0.001**
Medication	2.82 (1.10)	2.93 (0.97)	2.80 (1.01)	2.70 (1.13)	2.94 (1.36)	0.300
Interventions that target behavior such as increasing activity levels, meditation, etc.	3.33 (1.00)	**2.96 (0.76)** ^**a**^	**3.36 (0.97)** ^**b**^	**3.31 (1.09)** ^**b**^	**3.89 (0.99)** ^**c**^	**< 0.001**
Alternative treatments, like acupuncture	3.41 (1.08)	**3.05 (0.77)** ^**a**^	**3.52 (1.04)** ^**bc**^	**3.34 (1.23)** ^**ab**^	**3.92 (1.07)** ^**c**^	**< 0.001**

*Note:* Boldfaced values displayed omnibus significance at *p* < .00083. Different superscripts denote significant differences between groups according to post-hoc Games-Howell tests

Table 7 presents the prevalence of interest in various treatments as well as which treatments participants would refuse to try. In general, Group 4 (individuals with high pain-related facilitators but low social motives) exhibited the highest proportion of individuals who would be interested in various treatments, including acupuncture/acupressure; mindfulness/meditation/guided relaxation; massage; physical therapy/exercise; mental health therapy or counseling; nutrition-based programs; psychedelics; and herbal remedies. Group 3 (individuals with low behavior reduction outcome preferences) exhibited similarly high proportions for each of these therapies and had the highest proportion of interest for cannabis/marijuana; non-opioid medication; and patient education/information. Group 2 (individuals with high motivation and interest) typically exhibited lower prevalence of interest in each of these therapies. However, Group 1 (individuals with low pain-related facilitators) had the lowest prevalence of interest across all therapies.

**Table 7:** Differences in Treatment Interest and Refusal Among Motives and Preference Groups

			Group 1: Low Pain-Related Facilitators	Group 2: High Motivation and Interest	Group 3: Low Behavior Reduction Outcome Preference	Group 4: High Pain-Related Facilitators, Low Social Motives	
		Overall	n = 106	n = 112	n = 128	n = 66	*p*
Which of these treatments would you be **interested in trying**?							
	Acupuncture/Acupressure	48% (198)	**28% (30)**	**45% (50)**	**59% (76)**	**64% (42)**	**< 0.001**
	Yoga	32% (131)	22% (23)	34% (38)	35% (45)	38% (25)	0.072
	Tai Chi	23% (95)	13% (14)	23% (26)	25% (32)	35% (23)	0.010
	Mindfulness/ Meditation/ Guided relaxation	36% (150)	**15% (16)**	**42% (47)**	**43% (55)**	**48% (32)**	**< 0.001**
	Massage	44% (183)	**26% (28)**	**36% (40)**	**55% (71)**	**67% (44)**	**< 0.001**
	Cupping	19% (77)	9% (9)	21% (24)	19% (24)	30% (20)	0.003
	Physical therapy/ Exercise	41% (170)	**24% (25)**	**37% (41)**	**52% (67)**	**56% (37)**	**< 0.001**
	Chiropractic/ Adjustment/ Manipulation	26% (109)	18% (19)	24% (27)	27% (34)	44% (29)	0.002
	Mental health therapy or counseling (e.g., cognitive behavioral therapy; acceptance and commitment therapy)	25% (102)	**10% (11)**	**22% (25)**	**33% (42)**	**36% (24)**	**< 0.001**
	Nutrition-based program (e.g., diet or supplements)	33% (134)	**13% (14)**	**29% (32)**	**41% (52)**	**55% (36)**	**< 0.001**
	Psychedelics (e.g., psilocybin)	14% (59)	**4% (4)**	**11% (12)**	**18% (23)**	**30% (20)**	**< 0.001**
	Herbal remedies	33% (135)	**15% (16)**	**31% (35)**	**41% (53)**	**47% (31)**	**< 0.001**
	Cannabis/ Marijuana	25% (101)	**11% (12)**	**18% (20)**	**37% (47)**	**33% (22)**	**< 0.001**
	Non-opioid medication	26% (108)	**10% (11)**	**12% (14)**	**43% (55)**	**42% (28)**	**< 0.001**
	Opioid medication	18% (74)	16% (17)	11% (12)	23% (29)	24% (16)	0.047
	Patient education/ Information on pain conditions	20% (82)	**4% (4)**	**14% (16)**	**33% (42)**	**30% (20)**	**< 0.001**
What treatments would you **refuse to try**?							
	Acupuncture/Acupressure	7% (30)	7% (7)	5% (5)	9% (12)	9% (6)	0.500
	Yoga	12% (49)	10% (11)	12% (14)	12% (15)	14% (9)	> 0.9
	Tai Chi	10% (41)	11% (12)	6% (7)	12% (15)	11% (7)	0.500
	Mindfulness/ Meditation/ Guided relaxation	6% (24)	9% (9)	2% (2)	9% (11)	3% (2)	0.051
	Massage	4% (18)	9% (9)	3% (3)	5% (6)	0% (0)	0.041
	Cupping	14% (57)	**7% (7)**	**8% (9)**	**20% (26)**	**23% (15)**	**< 0.001**
	Physical therapy/ Exercise	4% (17)	6% (6)	2% (2)	6% (7)	3% (2)	0.400
	Chiropractic/ Adjustment/ Manipulation	12% (50)	**7% (7)**	**2% (2)**	**27% (34)**	**11% (7)**	**< 0.001**
	Mental health therapy or counseling (e.g., cognitive behavioral therapy; acceptance and commitment therapy)	7% (29)	9% (10)	3% (3)	9% (12)	6% (4)	0.120
	Nutrition-based program (e.g., diet or supplements)	3% (11)	4% (4)	1% (1)	3% (4)	3% (2)	0.600
	Psychedelics (e.g., psilocybin)	41% (169)	**18% (19)**	**47% (53)**	**52% (67)**	**45% (30)**	**< 0.001**
	Herbal remedies	8% (33)	**14% (15)**	**1% (1)**	**12% (15)**	**3% (2)**	**< 0.001**
	Cannabis/ Marijuana	35% (143)	40% (42)	44% (49)	27% (35)	26% (17)	0.014
	Non-opioid medication	10% (39)	15% (16)	14% (16)	4% (5)	3% (2)	0.002
	Opioid medication	28% (116)	**16% (17)**	**20% (22)**	**39% (50)**	**41% (27)**	**< 0.001**
	Patient education/ Information on pain conditions	4% (16)	6% (6)	2% (2)	6% (7)	2% (1)	0.300

*Note*: Boldfaced values displayed omnibus significance at *p* < .00083

Fewer differences emerged for refusal of various therapies. However, Group 4 (individuals with high pain-related facilitators) exhibited the highest prevalence of refusal for cupping and opioid medication. Group 3 (individuals with low behavior reduction outcome preference) exhibited the highest refusal of chiropractic/adjustment/manipulation and psychedelics. Overall, Group 2 (individuals with high motivation and interest) exhibited the lowest prevalence of refusal for chiropractic/adjustment/manipulation and herbal remedies.

## Discussion

Engaging individuals with chronic pain to participate in pain research is imperative for advancing the science of treatment. To our knowledge, there is a dearth of previous research exploring facilitators for research participation in the chronic pain population. The present study sought to advance our understanding of motives for hypothetical research participation and research outcome preferences as facilitators among individuals with self-reported chronic pain in order to better understand how to improve research representation. Our findings elucidated underlying dimensions of facilitation, as well as patient-centered profiles that may drive interest in research. Additionally, we found differences among these profiles in demographic and pain characteristics, willingness to participate in research, as well as treatment interest.

Motives for hypothetical research participation were grouped into three components, including social engagement/enjoyment; pain improvement/advancing science; and monetary compensation. Previous research has noted that many individuals are motivated to engage in research to help both others and themselves [8]. Our finding that pain improvement and advancing science are strong facilitators is supported by previous research with individuals with chronic pain, which found that patients often cited both motives of altruism (i.e., contributing to research) and personal benefit (i.e., seeking relief from pain) [7]. Previous research also supports the desire for compensation as a motive for research participation [7, 18]; however, this was seldom indicated as a motive for research participation in our sample of individuals with self-reported chronic pain. To our knowledge, previous research has not shown facilitation by social motives and enjoyment of the research process. This dimension should be further explored in future research. Although some individuals did not rate this domain highly, this may be a fruitful component to tap into for certain individuals to increase research participation.

We additionally found that research outcome preferences were grouped into three components, including co-occurring symptom reduction; behavior reduction modification; and pain and function improvement. Not surprisingly, patients who valued research focused on improving their pain and physical functioning also tended to value research that sought to improve co-occurring symptoms, such as depression and sleep-related problems. This exemplifies the broad impact that chronic pain can have on other aspects of life and indicates that chronic pain patients want researchers to focus on improving these aspects, in addition to improving pain. The importance of pain, functioning, and co-occurring symptom improvement is supported by previous research that suggests pain reduction, enjoyment of life, emotional well-being, fatigue, weakness, and sleep-related problems were areas of interest most important to the chronic pain population [9, 10]. Research interest related to behavior modification such as reducing or stopping medication use or behaviors such as drinking or smoking may be of interest for some individuals but may dissuade others from participating. For some, such behavior modification may not be relevant. This is a new and potentially important factor that should be explored in future research.

The findings suggest four potential participant-centered profiles utilizing these dimensions of facilitators. Interestingly, these four groups did not differ significantly in pain severity, physical function, anxiety symptoms, or perceived health. However, differences emerged in demographic characteristics, depressive symptoms, research participation willingness, and treatment interest.

Group 1 (individuals with low pain-related facilitators) rated each of the facilitators relatively low compared with the other groups. This group also generally expressed relatively low interest in the different treatments and types of studies proposed to them. Group 1 also tended to be higher in depressive symptoms than the other three groups. Taken together, these findings suggest that a feeling of hopelessness may be the cause of this group’s low motivation for research participation. Those with chronic pain who are also depressed may be more challenging to recruit for research studies, which is a significant issue given the frequent co-occurrence of chronic pain and depression [19]. Indeed, a recent review reported that depressive symptoms impact individuals’ willingness to participate in clinical trials, with hesitance amplified, for example, by the risk of the study to mental health and embarrassment or discomfort with study-related procedures [2].

Group 2 (individuals with high motivation and interest) rated all motives and outcome preferences relatively high compared to the other groups. However, they tended to rate interest in different types of studies and treatments relatively low. It is unclear why this discrepancy was found, but it indicates self-reported facilitators may not correspond to actual willingness in this group of individuals with self-reported chronic pain. We hypothesize it may be that barriers to research participation were incorporated into estimations of willingness, whereas barriers may not have been considered when judging motives and research outcome preferences. Of note, both Groups 1 and 2 tended to be younger, more often male, less often white, more often employed, and had pain of shorter duration. These characteristics may present unique barriers to research participation. For example, the higher proportion of minorities in these two groups may correspond to higher rates of mistrust of medical research, potentially explaining their low willingness [20, 21]. Their low willingness to participate and try different treatments may also be because individuals from these groups were more often employed than the others, making it difficult to find time for research participation and pain therapies. In contrast to Group 1, however, the relatively high facilitator scores suggest Group 2 may be persuaded to participate in research if they saw the opportunity for personal or societal benefit and barriers to participation could be ameliorated.

Groups 3 (individuals with low behavior reduction outcome preference) and 4 (individuals with high pain-related facilitators but low social motives) were similar demographically, tending to be older, more often female, more often white, more often unemployed, and having pain of longer duration. Group 3 did not score particularly high on any motive or outcome preference but showed comparatively low interest in treatments that focused on behavior reduction modification. Indeed, Casarett et al. [10] found the desire for research to examine reducing medication dosage was associated with being younger. Group 3 was, however, relatively willing to participate in different types of studies and try different treatments compared to Group 1 and 2. This group’s low motivation for, yet willingness to participate in, research indicates these individuals may merely need to be presented with the opportunity to participate in research, but they may not seek out research participation. This highlights the need for active recruitment approaches instead of passive methods.

Group 4 (individuals with high pain-related facilitators but low social motives) was the most willing of the four groups to participate in the various treatments and study types that were presented to them. Of note, they were not particularly motivated by social engagement and enjoyment of the research process. However, they were motivated by pain and functioning improvement, co-occurring symptom reduction, and advancing science. The fact that Groups 3 and 4 tended to be older and have pain of longer duration is noteworthy, and potentially explains their willingness to try a variety of treatments and study types. This population likely has exhausted other treatment options for chronic pain, and therefore may be drawn to research studies exploring new or unconventional treatments.

### Research implications

Knowing the motivations and preferred outcomes of individuals with self-reported chronic pain can help improve recruitment and retention in clinical studies, thus leading to new discoveries and improved clinical care. We recommend appealing to potential research participants’ desire to advance science and improvement of their pain and co-occurring symptoms during recruitment. However, research staff should be mindful of the potential vulnerability of individuals with chronic pain who are seeking relief. It must be made clear to potential participants during the informed consent process that pain and co-occurring symptom relief is not guaranteed if they participate. Additionally, the study structure should be clearly conveyed, such as the possibility that some individuals may be assigned to a ‘usual care’ arm. Studies should implement sound informed consent procedures such as providing information in various ways (i.e., orally, in writing, and graphics for complex trials) and implementing a comprehension assessment using open-ended questions. Some with self-reported chronic pain are also motivated by the opportunity for social engagement, which may be another angle to approach from. This may be particularly important for individuals who are traditionally harder to recruit (e.g., younger, employed).

Unfortunately, there exists a subgroup of those with self-reported chronic pain that are less motivated and willing to participate in research than others (Group 1), and this group tends to have higher depressive symptoms and pain of shorter duration. This group also tends to be non-white, which may pose issues for recruiting a diverse sample. Future research should focus on successful recruitment strategies in this group, such as intensified efforts to reduce barriers, increase trust in the study team, and potentially appeal to the possibility for hope. Again, however, researchers should be cautious to not make unsubstantiated promises about the research itself, such as promises of symptom improvement; such practices will likely increase mistrust among study participants. However, there also exist groups of individuals who are highly motivated to participate in research (Groups 2 and 4) and willing to try many different types of treatments and research studies (Groups 3 and 4). The individuals in these groups likely make up the majority of the current research participant population. Yet, certain efforts may improve recruitment such as lowering barriers (Group 2) or intensified and active advertising campaigns (Group 3). Our data suggest there are certain individuals who are interested in different types of treatments and research, which may present bias into these studies. For example, individuals that have had pain for longer duration tend to be the most willing to participate in various types of studies and treatments. Additionally, older individuals may be less interested in research that examines methods to reduce their medication dosage. Concerted efforts should be made to recruit individuals with other facilitators in order to improve diversity and generalizability.

### Limitations

There are a number of limitations that should be considered. First, motives and outcome preferences were gauged from survey responses. Our questionnaire did not select for people currently participating in research or evaluate future research enrollment, so all answers were hypothetical. The difference between being willing to participate in research and actually doing so is important. Satisfaction with past research participation may also impact willingness to participate in future research [22], which was not accounted for in the present study.

Our sample was also limited. Individuals self-reported chronic pain; however, we do not have information on pain diagnoses, location of pain, or pain origin. Another limitation is that our survey was completed only by individuals in the state of Michigan. Participants’ perspectives on research may change based on the location of research studies in different areas of the world and the community being targeted. Participants in our study were compensated for their time; yet, many indicated that compensation was not a strong motive for hypothetical research participation. It is possible that participants were responding in a socially desirable way. However, it is also possible that compensation, though studies may provide it, may not be rated as highly as other motives for participation. It should also be noted that the participants in the present study demonstrated they are willing to participate in research in some form simply through the completion of our study survey. It is likely that many individuals with chronic pain would not participate in research of any kind, and it is therefore impossible to study this group and understand what would motivate them to participate in research or what research outcomes they desire. Further, the requirements and incentives of participation in a given study may impact what motivates an individual to participate, and this was not accounted for in our survey.

Our measures also presented limitations. We necessarily included a limited set of potential motives and research outcome preferences. Yet, these are not established scales and additional factors may be important for participants. Lastly, our choice of analytic methods (i.e., principal components analysis and latent profile analysis) rely to a certain degree on the researchers’ judgment and interpretation. Thus, future research should attempt to replicate the current findings.

## Conclusion

Our data provide a framework of potential motives for hypothetical research participation and research outcome preferences that researchers can use to improve participation in chronic pain research trials. It also gives an indication of who may respond best to advertisements and recruitment strategies that appeal to a given motive or preferred outcome. Researchers should use this data to improve recruitment for their studies and to monitor any potential biases in their subject samples.

### Electronic supplementary material

Below is the link to the electronic supplementary material.

**Supplementary Material 1: Supplemental Figure 1**. Recruitment Strategy and Timeline. **Supplemental Table 1**. Wave I Social Medial Recruitment Sites. **Supplemental Table 2**. Wave II Veteran Social Media Sites. **Supplemental Table 3**. Wave III Targeted Social Media Recruitment Cities

**Supplementary Material 2: Supplemental Table 4**. Checklist for Reporting Of Survey Studies (CROSS)

**Supplementary Material 3**: **Supplemental Table 5**. Bivariate Correlations Among Motives for Research Participation Items. **Supplemental Table 6**. Bivariate Correlations Among Research Outcome Preferences Items

## Data Availability

Data, analysis code, and output are available on Open Science Framework (https://osf.io/8m5gs/?view_only=f0637c6e9b4748209f5d6a67f1652355).
