# Supplementary material for: Understanding facilitators of research participation among adults with self-reported chronic pain – a survey examining hypothetical research participation
Source: BMC Med Res Methodol. 2024 Jan 22;24:18. doi: 10.1186/s12874-023-02128-8 (PMC10802039; doi:10.1186/s12874-023-02128-8)
Supplement: Supplementary file 1 — Supplementary Material 1: Supplemental Figure 1. Recruitment Strategy and Timeline. Supplemental Table 1. Wave I Social Medial Recruitment Sites. Supplemental Table 2. Wave II Veteran Social Media Sites. Supplemental Table 3. Wave III Targeted Social Media Recruitment Cities [file 12874_2023_2128_MOESM1_ESM.pdf]

Supplemental Figure 1. Recruitment Strategy and Timeline

January 21, 2022 (Wave I):
- Began recruitment. Posted advertisement and survey link to chronic pain social media sites (see Supplemental Table 1 below).

↓

February 7, 2022:
- Initiated advertisement campaign through University of Michigan social media sites in collaboration with Michigan Institute for Clinical and Health Research (MICHR).

↓

February 18, 2022:
- Paused MICHR social media recruitment due to low response.

↓

March 3, 2022 (Wave II):
- Recognized low rate of male recruitment.
- Sent survey to veteran social media sites (see Supplemental Table 2 below).

↓

March 25, 2022 (Wave III):
- Recognized low rate of non-White recruitment.
- Developed list of Michigan cities with high diversity using census data and sorting by:
  - Highest population
  - Median income under 55k USD
  - Lowest percentage of White population
- Identified social media sites related to community centers, clinics, and libraries within the top 30 cities identified using the qualities described above (see Supplemental Table 3 below).

↓

March 28, 2022:
- Reinitiated MICHR social media recruitment campaign to encourage participation from individuals identifying as Black, Indigenous, or People of Color (BIPOC).

↓

March 29, 2022:
- Updated the language used in our social media message to encourage participation from individuals identifying as Black, Indigenous, or People of Color (BIPOC).

↓

April 5, 2022:
- Ended MICHR social media recruitment.

↓

April 28, 2022:
- Ended recruitment.

Supplemental Table 1. Wave I Social Medial Recruitment Sites

The forum name indicates the social media site that was targeted in the first wave of recruitment and the pain condition that is relevant to that social media site. Advertisements for the survey study were posted on these social media sites for potential participants to access.

**Forum Name:**	**Pain Condition:**
Arthritis and joint disease	Arthritis and joint pain
Back & Joint Pain Treatment	Back and Joint pain
Back Pain	Back Pain
Celiac Disease|Michigan	Celiac Disease
Chronic back pain support group	Back Pain
Chronic Daily Headache and Migraine Support Group	Headaches
Chronic Fatigue Syndrome / Myalgic Encephalomyelitis	Chronic Fatigue Syndrome
Chronic Fatigue Syndrome / Myalgic Encephalomyelitis	Chronic Fatigue Syndrome
Chronic Fatigue Syndrome /M.E - Increase awareness & understanding!	Chronic Fatigue Syndrome
CHRONIC FATIGUE SYNDROME/FIBROMYALGIA	Chronic Fatigue Syndrome and Fibromyalgia
CHRONIC FATIGUE SYNDROME/FIBROMYALGIA (CFS/FM) IS TREATABLE	Chronic Fatigue Syndrome and Fibromyalgia
Chronic Headaches: Awareness & Support	Headaches
Chronic Pain	Chronic pain in general
Chronic Pain Management Group	Chronic pain in general
Chronic Pain Support Group	Chronic pain in general
Chronic Pain Treatment Support Group	Chronic pain in general
Chronic Pain...The Crippling Disease	Chronic pain in general
Complex Regional Pain Syndrome	Complex Regional Pain Syndrome
Complex Regional Pain Syndrome (CRPS) Help & Support Group	Complex Regional Pain Syndrome
Endometriosis Support Group	Endometriosis
Fibromyalgia - An Optimistic but Realistic Support Group	Fibromyalgia
Fibromyalgia and Chronic Pain	Fibromyalgia and chronic pain
fibromyalgia support	Fibromyalgia
IBS-C, IBS-D, IBS-M Support Group	Irritable Bowel Syndrome
Interstitial Cystitis - News, Information, Advice, Support	Interstitial Cystitis
INVISIBLE DISEASES .com* Support Chronic Fatigue Syndrome /ME, Fibro & Lyme	Chronic Fatigue Syndrome, Fibromyalgia & Lyme Disease
LIVING IN CHRONIC PAIN	Chronic pain in general
Michigan Chronic Pain Forum	Chronic pain in general
Michigan Chronic Pain Survivors	Chronic pain in general
Michigan Endometriosis Support	Endometriosis
Michigan Spoonies; chronic illness/pain/fatigue & invisible disabilities	A bunch of different chronic illnesses
Mid-Michigan Women's Fibromyalgia and Chronic Illness SupportGroup	Fibromyalgia
Rheumatoid Arthritis	Rheumatoid Arthritis
RSD/CRPS and Neuropathic pain syndrome	Complex Regional Pain Syndrome

Supplemental Table 2. Wave II Veteran Social Media Sites

These indicate the social media sites established for military veterans on which advertisements for the survey study were posted for potential participants to access.

Veterans News and Information
Veterans with Chronic Pain

Supplemental Table 3. Wave III Targeted Social Media Recruitment Cities

These are Michigan cities meeting criteria (See Supplemental Figure 1) for targeted recruitment efforts. Social media sites linked to community centers, clinics, and libraries were identified in these cities. Advertisements for the survey study were then posted on these sites for potential participants to access.

Auburn Hills city, Michigan
Battle Creek city, Michigan
Detroit city, Michigan
East Lansing city, Michigan
Eastpointe city, Michigan
Farmington Hills city, Michigan
Flint city, Michigan
Grand Rapids city, Michigan
Hamtramck city, Michigan
Harper Woods city, Michigan
Holland city, Michigan
Inkster city, Michigan
Jackson city, Michigan
Kalamazoo city, Michigan
Kentwood city, Michigan
Lansing city, Michigan
Mount Clemens city, Michigan
Muskegon city, Michigan
Oak Park city, Michigan
Pontiac city, Michigan
Romulus city, Michigan
Roseville city, Michigan
Saginaw city, Michigan
Sault Ste. Marie city, Michigan
Southfield city, Michigan
Taylor city, Michigan
Warren city, Michigan
Wayne city, Michigan
Westland city, Michigan
Wixom city, Michigan
Ypsilanti city, Michigan
