# Supplementary material for: Understanding facilitators of research participation among adults with self-reported chronic pain – a survey examining hypothetical research participation
Source: BMC Med Res Methodol. 2024 Jan 22;24:18. doi: 10.1186/s12874-023-02128-8 (PMC10802039; doi:10.1186/s12874-023-02128-8)
Supplement: Supplementary file 3 — Supplementary Material 3: Supplemental Table 5. Bivariate Correlations Among Motives for Research Participation Items. Supplemental Table 6. Bivariate Correlations Among Research Outcome Preferences Items [file 12874_2023_2128_MOESM3_ESM.docx]

| Supplemental Table 5. Bivariate Correlations Among Motives for Research Participation Items | | | | | | | | | | | | | | |
| --- | --- | --- | --- | --- | --- | --- | --- | --- | --- | --- | --- | --- | --- | --- |
|  |  |  |  | | |  | | |  |  |  |  |  |  |
| I would participate in research... | | 1 | 2 | 3 | 4 | 5 | 6 | 7 | 8 | 9 | 10 | 11 | 12 | 13 |
| 1 | To improve the wellbeing of society. | -- |  |  |  |  |  |  |  |  |  |  |  |  |
| 2 | To improve my quality of life and health. | .50 | -- |  |  |  |  |  |  |  |  |  |  |  |
| 3 | To learn about my pain. | .41 | .51 | -- |  |  |  |  |  |  |  |  |  |  |
| 4 | To have access to new treatments. | .34 | .55 | .57 | -- |  |  |  |  |  |  |  |  |  |
| 5 | To have faster and more available access to doctors. | .25 | .42 | .42 | .51 | -- |  |  |  |  |  |  |  |  |
| 6 | To receive medical care without insurance/payment. | .13 | .19 | .19 | .29 | .40 | -- |  |  |  |  |  |  |  |
| 7 | To receive compensation. | .06 | .10 | .21 | .24 | .29 | .46 | -- |  |  |  |  |  |  |
| 8 | If it was recommended by my doctor. | .21 | .27 | .32 | .37 | .33 | .32 | .29 | -- |  |  |  |  |  |
| 9 | If it was recommended by family or friends. | .06 | .12 | .23 | .29 | .37 | .26 | .33 | .55 | -- |  |  |  |  |
| 10 | If it’s easy for me to do. | .24 | .35 | .34 | .39 | .29 | .13 | .28 | .35 | .37 | -- |  |  |  |
| 11 | If a lot of people I know do it. | -.11 | -.12 | .08 | .07 | .18 | .25 | .34 | .31 | .50 | .21 | -- |  |  |
| 12 | If it is fun. | .07 | .05 | .19 | .15 | .20 | .26 | .38 | .24 | .41 | .38 | .56 | -- |  |
| 13 | To share my story. | .12 | .14 | .26 | .21 | .20 | .22 | .27 | .24 | .33 | .24 | .38 | .49 | -- |
| 14 | To fill my time. | -.13 | -.14 | .00 | .02 | .14 | .22 | .30 | .23 | .40 | .14 | .51 | .50 | .45 |

| Supplemental Table 6. Bivariate Correlations Among Research Outcome Preferences Items | | | | | | | | | | | | | |
| --- | --- | --- | --- | --- | --- | --- | --- | --- | --- | --- | --- | --- | --- |
|  |  |  |  | | |  | | |  |  |  |  |  |
| How important do you consider the following outcomes: | | 1 | 2 | 3 | 4 | 5 | 6 | 7 | 8 | 9 | 10 | 11 | 12 |
| 1 | Lower severity or intensity of pain | -- |  |  |  |  |  |  |  |  |  |  |  |
| 2 | Better physical functioning | .59 | -- |  |  |  |  |  |  |  |  |  |  |
| 3 | Learning new ways to manage pain | .56 | .50 | -- |  |  |  |  |  |  |  |  |  |
| 4 | Increased frequency of healthy behaviors like exercise | .38 | .43 | .47 | -- |  |  |  |  |  |  |  |  |
| 5 | Decreased frequency of behavior like drinking or smoking | .01 | .08 | .16 | .25 | -- |  |  |  |  |  |  |  |
| 6 | Better mood (like lower depression or anxiety symptoms) | .38 | .38 | .33 | .32 | .33 | -- |  |  |  |  |  |  |
| 7 | Better sleep | .46 | .52 | .43 | .35 | .20 | .52 | -- |  |  |  |  |  |
| 8 | Less fatigue | .48 | .57 | .40 | .31 | .10 | .45 | .57 | -- |  |  |  |  |
| 9 | Better mental clarity and concentration | .35 | .40 | .40 | .33 | .19 | .47 | .47 | .53 | -- |  |  |  |
| 10 | Increased ability to enjoy life | .51 | .49 | .43 | .39 | .16 | .49 | .49 | .54 | .55 | -- |  |  |
| 11 | Better relationships, such as with friends or a romantic partner | .21 | .27 | .29 | .32 | .35 | .50 | .40 | .34 | .55 | .50 | -- |  |
| 12 | Better overall well-being | .51 | .50 | .46 | .34 | .19 | .49 | .55 | .50 | .51 | .62 | .49 | -- |
| 13 | Reducing or stopping a medication | .12 | .27 | .30 | .27 | .31 | .26 | .27 | .26 | .38 | .29 | .45 | .27 |
